# More bang for your buck: Super-adiabatic quantum engines

**DOI:** 10.1038/srep06208

**Published:** 2014-08-28

**Authors:** A. del Campo, J. Goold, M. Paternostro

**Affiliations:** 1 Theoretical Division, Los Alamos National Laboratory, Los Alamos, NM 87545, USA; 2 Center for Nonlinear Studies, Los Alamos National Laboratory, Los Alamos, NM 87545, USA; 3 The Abdus Salam International Centre for Theoretical Physics, 34014, Trieste, Italy; 4 Centre for Theoretical Atomic, Molecular and Optical Physics, School of Mathematics and Physics, Queen's University, Belfast BT7 1NN, United Kingdom

## Abstract

The practical untenability of the quasi-static assumption makes any realistic engine intrinsically irreversible and its operating time finite, thus implying *friction* effects at short cycle times. An important technological goal is thus the design of maximally efficient engines working at the maximum possible power. We show that, by utilising shortcuts to adiabaticity in a quantum engine cycle, one can engineer a thermodynamic cycle working at finite power and zero friction. Our findings are illustrated using a harmonic oscillator undergoing a quantum Otto cycle.

THermodynamics is the study of heat and its interconversion to mechanical work. It successfully describes the “equilibrium” properties of macroscopic systems ranging from refrigerators to black holes^1^. The revolution potentially embodied by the implementation of quantum technologies is motivating the consideration of quantum devices going all the way down to the micro- and nano-scale^2^. This has forced us to revise our interpretation of thermodynamics to include *ab initio* both quantum and thermal fluctuations. The former, indeed, become quite prominent at such scales. In fact, far from equilibrium, quantum fluctuations become dominant and cannot be neglected. In turn, thermodynamic quantities such as work and heat become inherently stochastic and should be reformulated accordingly.

Recently discovered work fluctuation theorems (FTs) and the corresponding framework, which is known to hold both in quantum and classical systems, are extremely useful for the task of setting up a quantum apparatus for thermodynamics^3^,^4^,^5^,^6^,^7^. FTs set fundamental constraints on the energy fluctuations of a general thermodynamic system and embody useful tools to understand the thermodynamic implications of finite-time transformations^8^,^9^. Whether or not FTs can help us shedding light on the limitations or possible advantages of a quantum device operating in finite-time is a very important point to address, that goes beyond the scopes of this paper. However, it is sensible to expect that a convenient platform for the provision of quantitative answers in this sense could come from the study of quantum engines, for which the thermodynamic laws must be recast appropriately^10^,^11^,^12^,^13^. In fact, although the working principles of a reversible engine might well be quantum mechanical, its efficiency would always be limited by the second law, thus making the quantum version of cycles similar to their classical counterparts.

The assumed reversibility (quasi-stationarity) of an engine cycle, which implies an infinitely long cycle-time, determines its inevitable zero-power nature. This clearly crashes with the reality of any practical machine, either quantum or classical. However, the finite-time operation of a machine working in finite-time exposes it to the effects of friction-induced losses. The key engineering goal in this context is thus to find the maximum efficiency allowed at the maximum possible power^15^,^16^. Noticeably, in Ref. 17 an ultra-fast cycle has been discussed that nevertheless attains Carnot efficiency.

Classical approaches to the maximization of the power output of thermal engines have been proposed and developed in the past^18^,^19^. While Ref. 14 proposes the use of systematic noise to suppress friction in the expansion and compression stages of a quantum Otto cycle, here we devise an innovative way to run a finite-time, finite-power quantum cycle based on the use of quantum shortcuts to adiabaticity^20^,^21^,^22^,^23^,^24^. Such techniques have been employed to show that the Hamiltonian of a quantum system can be manipulated in a way to mimic an adiabatic process via a non-adiabatic shortcut^20^,^21^,^22^,^23^,^24^. In this context, the term “adiabatic” should be interpreted as “slow” and will be used, unless the context is evidently different (such as in the description of the cycle), as synonymous of “quasi-static”. A few experiments have demonstrated several of such proposals^25^,^26^,^27^. In this paper we show that such Hamiltonian-engineering techniques allow us to drive the expansion and compression stages of a cycle, which are prone to frictional effects, virtually without any loss affecting the performance of a quantum engine within the finite-time of its cycle. Inspired by a recent ion-trap proposal^13^, we will provide an example of a finite-time, fully frictionless quantum Otto cycle where the working medium is a quantum harmonic oscillator. Remarkable examples of recent works along the lines of our investigation have been reported recently^28^,^29^, although they both address classical analogs of quantum shortcuts to adiabaticity with Deng *et al.* studying the performance of an Otto cycle from such perspective^29^.

## Results

**Figure 1:**
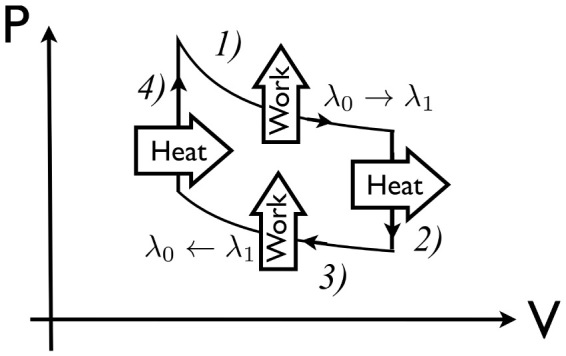
Pressure-volume diagram of a (quantum) Otto cycle. The numbers relate the processes to the description of each step given in the main text. We identify the steps where heat enters (exits) the working medium and those where work is performed by (done onto) it as a result of a corresponding change in the work parameter *λ* (*t*).

**Quantum Otto Cycle.** In an Otto engine, a working medium (coupled alternatively to two baths at different temperatures *T_i_*, *i* = 1, 2) undergoes a four-stroke cycle. In its quantum version, the state of the working medium is described by a density operator *ρ*(*λ*(*t*)) that is changed by the Hamiltonian 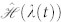. Here, *λ*(*t*) is a *work parameter*, typical of the specific setting used to implement 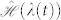, whose value determines the equilibrium configuration of the system. As illustrated in Fig. 1, the cycle steps are as follows:

1. *An adiabatic expansion* performed by the change *λ*_0_ ≡ *λ*(0) → *λ*_1_ ≡ *λ*(*τ*_1_), where *τ*_1_ is the time at which this step ends. As a result of this transformation, work is extracted from the medium due to the change in its internal energy.
2. *A cold isochore* where heat is transferred from the working medium to the cold bath. This is associated with a heat flow from the medium to the cold reservoir.
3. *An adiabatic compression* performed by the reverse change of the work parameter *λ*_1_ → *λ*_0_ and during which work is done on the medium.
4. *A hot isochore* during which heat is taken from the hot reservoir by the working medium.

If the engine is run in a finite-time, *i.e.* we abandon the usual quasi-static assumption, friction is generated along the expansion and compression steps. We will elucidate the nature of this friction later on. In addition, one may (realistically) assume imperfect heat conduction during the isochores. Under such conditions, the work *W* done by/on the engine and the heat *Q* exchanged by the medium with the baths, become stochastic quantities. The efficiency of the engine is then defined as the ratio between the average total work per cycle and the average heat received from the hot bath, that is 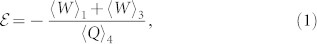
where 〈*K*〉*_j_* (for *K* = *Q*, *W*) is evaluated during step *j* = 1, …, 4. The power of the engine is then 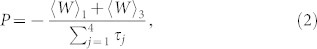
where *τ_j_* is the time needed for step *j*. Here we consider the case where friction only occurs along the adiabatic transformations and neglect fluctuations in the heat flow. On the other hand, we should not forget about the thermalisation process inherent in the isochores, which are associated with the production of entropy. We thus assume to have identified a regime such that the entropy produced by such thermalisation steps is negligible and associated with finite values of *τ*_2,4_ (see Supplementary Information for the determination of such a working point and an estimate of both the order of magnitude of such time intervals and of the corresponding production of entropy). Needless to say, the entropy produced during the isochores is the same regardless of the way we perform the adiabats. Moreover, as we will describe in the second part of this paper, the strategy based on shortcuts to adiabaticity that we propose makes 〈*W*〉*_i_* independent of *τ_i_*. In these conditions, the maximisation of Eq. (2) is achieved by minimizing *τ*_1,3_. We now describe our protocol to reduce the time needed for the adiabats and keep the associated friction at bay.

*Finite-time thermodynamics*. Before we quantify the efficiency of the engine, we need to define the probability distribution of work of which 〈*W*〉 is the first moment. We consider a Hamiltonian 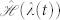 applied to a system prepared into the Gibbs state 
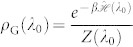
. with inverse temperature *β* and *λ*(*t* ≤ 0) = *λ*_0_. Here, 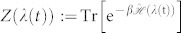 is the partition function. At *t* = 0, the system-reservoir coupling is removed and *λ* is changed from its initial value *λ*_0_ to *λ*_1_ at *t* = *τ*. Such process could be the expansion/compression step of the Otto cycle, represented by a change of *λ* in 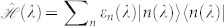, where |*n*(*λ*)〉 is the *n*^th^ eigenstate with eigenvalue *ε_n_*(*λ*). In this context, work should be reformulated in a way to account for both the statistics of the initial state of the system and the non-deterministic nature of quantum measurements^30^. Under the assumption 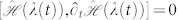, the corresponding work distribution *P*(*W*;*t*) reads 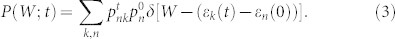
Here we use the notation shortcut |*n*(*t*)〉 = |*n*(*λ*(*t*))〉, and call 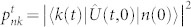 the probability that, under the action of the evolution operator 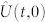 associated with 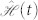, the system goes from the initial state |*n*(0)〉 to the final one |*k*(*t*)〉. Finally, 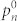 is the occupation probability of the initial state |*n*(0)〉, which for a Gibbs ensemble reads 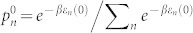. For 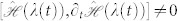, this expression needs to be modified^31^. However, regardless of the value of such commutator, the first two moments of the work distribution read 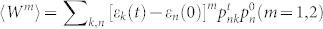.

For finite systems, the statistical nature of work requires the second law of thermodynamics to be revised to 〈*W*〉 ≥ Δ*F*, with Δ*F* the change in free energy and the equality holding for a quasi-static isothermal process (the inequality holding strictly for all quasi-static processes performed without the coupling to a thermal reservoir). For non-ideal processes, the deficit between 〈*W*〉 and Δ*F* can be accounted for by the introduction of the average irreversible work 〈*W*_irr_〉 as 〈*W*〉 = 〈*W*_irr_〉 + Δ*F*. The behavior of 〈*W*〉 will be later compared with the average work 〈*W*_ad_〉 performed onto (or made by) the system in an adiabatic process. For such quantity, in the absence of a heat bath, we have 〈*W*_ad_〉 > Δ*F*. For a closed quantum system, the incoming heat flow is null and the irreversible entropy is Δ*S*_irr_ = *β*(〈*W*〉 − Δ*F*) = *β*〈*W*_irr_〉, which can be recast as 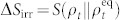
32 with *S*(*ρ_A_*||*ρ_B_*) = Tr(*ρ*_A_ln*ρ*_A_ − *ρ*_A_ln*ρ*_B_) the relative entropy between two density matrices *ρ_A_* and *ρ_B_*^33^, *ρ_t_* the time-evoluted state, and 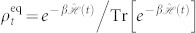 the corresponding equilibrium state at the temperature 1/*β*. Here, 〈*W*_irr_〉 quantifies the degree of friction caused by the finite-time protocol on the expansion or compression stage of the engine cycle. When a bath is reconnected, such friction results in dissipation and hence the decrease in the overall efficiency of the motor. For the point of demonstration we allow only this form of irreversibility in our cycle, although in principle the same analysis can be done for fluctuating heat flows^34^,^35^.

**Friction-free finite-time engine.** Recently, substantial work has been devoted to the design of super-adiabatic protocols, *i.e.* shortcuts to states which are usually reached by slow adiabatic processes^20^,^21^,^22^,^24^. A typical approach for shortcuts to adiabaticity is to use *ad hoc* dynamical invariants to engineer a Hamiltonian model that connects a specific eigenstate of a model from an initial to a final configuration determined by a dynamical process. Here we will rely on an approach based on engineered non-adiabatic dynamics achieved using self-similar transformations^23^,^36^.

Let us consider a quantum harmonic oscillator with time-dependent frequency *ω*(*t*) as the working medium of the engine cycle^23^. The Hamiltonian model that we consider is thus 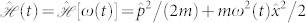, where 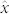 and 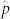 are the position and momentum operators of an oscillator of mass *m*. Inspired by the scheme in Ref. 13, we will use the tuneable harmonic frequency to implement the compression and expansion steps of the Otto cycle. In line with such proposal, the frequency of the harmonic trap embodies the volume of the chamber into which the working medium is placed, while the corresponding pressure is defined in terms of the change of energy per unit frequency.

Clearly, in the compression or expansion stage of the Otto cycle, the frequency of the trap will have to be varied, so that *ω*(*t*) takes here the role of a work parameter. We now suppose to subject the working medium to a change in the work parameter occurring in a time *τ* and corresponding to one of the friction-prone steps of the Otto cycle. Our goal is to design an appropriate shortcut to adiabaticity to arrange for a fast, frictionless evolution between the configurations of the working medium at *t* = 0 and that at *t* = *τ*. In order to do this, we remind that the wavefunction *ϕ_n_*(*x*, *t* = 0) = 〈*x*|*n*(0)〉 of an initial eigenstate |*n*(0)〉 of 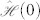 is known to follow the self-similar evolution^23^

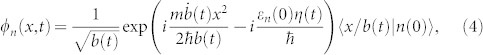
where 
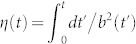
, *ε_n_*(0) is the energy of the eigenstate being considered at *t* = 0, and the scaling factor *b* is the solution of the Ermakov equation 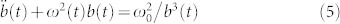
with the initial conditions *b*(0) = 1 and 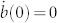. Needless to say, while the physically relevant parameter is the time-dependent frequency *ω*(*t*), the determination of the exact scaling parameter *b*(*t*) is key for the engineering of the correct shortcut to adiabaticity. This is found by inverting the Ermakov equation and complementing the previous set of boundary conditions with 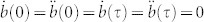, and 
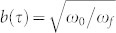
 with *ω*_0_ = *ω*(0) and *ω_f_* = *ω*(*τ*). An instance of the solution to this problem can be found in the Methods, where we give the explicit form of *b*(*t*) such that the finite-time dynamics taking the initial state *ϕ_n_*(*x*, *t* = 0) = 〈*x*|*n*(0)〉 to the final one 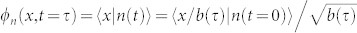 mimics the wanted adiabatic evolution (albeit for any *t* ∈ (0, *τ*), *ϕ_n_*(*x*, *t*) is in general different from the eigenstate |*n*(*t*)〉 of 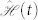). The choice of a harmonic oscillator is not a unique example as analogous self-similar dynamics can be induced in a large family of many-body systems^36^ and other trapping potentials, such as a quantum piston^37^. The resilience of the shortcuts to adiabaticity approach to imperfections in the engineering of the exact functional form of the time-dependent protocol embodied by *ω*(*t*) is an important point to address. Overall, shortcuts to adiabaticity are known to be robust against perturbations, as discussed in Ref. 23 for the case of an approximately harmonic trap and in Ref. 36 for other trapping potentials.

Let us consider the fluctuations induced in the expansion and compression stages of the Otto cycle when the above shortcut to adiabaticity is implemented. Let us consider a driving Hamiltonian with instantaneous eigenstates |*n*(*t*)〉 and eigenvalues *ε_n_*(*t*). In the adiabatic limit, the corresponding transition probabilities 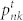 tend to |〈*n*(*t*)|*k*(*t*)〉|^2^ = *δ_k_*_,*n*_(*t*) for all *t* ∈ [0, *τ*]. The average work is then 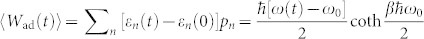. On the other hand, in a shortcut to adiabaticity, only the weaker condition 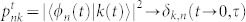 holds. For the time-dependent harmonic oscillator, it follows that 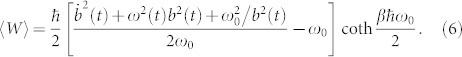
In the adiabatic limit 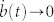 and 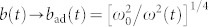.

**Figure 2:**
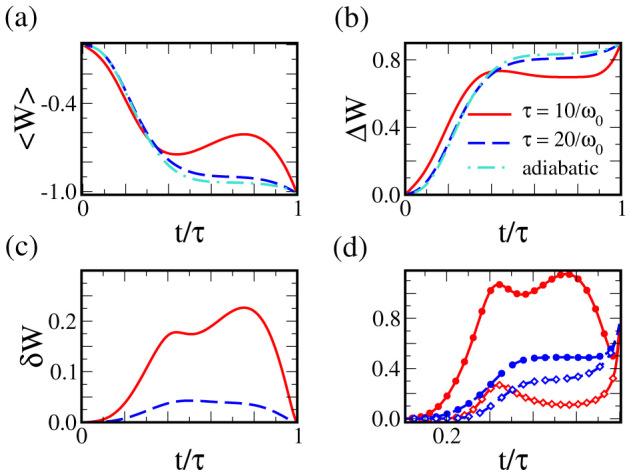
Work fluctuations along a shortcuts to an adiabaticity expansion. (a) Average work; (b) Standard deviation of the work; (c) Non-equilibrium deviations from the adiabatic average mean work; (d) We show 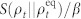 (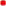 and 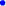) and 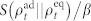 (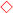 and 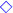) [cf. Eq. (7)] for the same processes shown in the other panels. All quantities are plotted in units of ħ*ω*_0_(*β* = 1).

Fig. 2(a) shows 〈*W*〉 along a shortcut to an adiabatic expansion in comparison with the corresponding adiabatic process 〈*W*_ad_(*t*)〉 (the behavior observed during a shortcut to a compression is mirrored in time). We stress that 〈*W*〉 is the work done on either adiabat until the reconnection with the bath, i.e. just prior to the isochoric heating/cooling stage. Fig. 2(b) displays the standard deviation Δ*W* = [〈*W*^2^〉 − 〈*W*〉^2^]^1/2^, which provides a further characterisation of the work fluctuations along the shortcut through the width of *P*(*W*;*t*). Interestingly, upon completion of the stroke, the non-equilibrium deviation of both the average work and the standard deviation from the adiabatic trajectory disappear.

We shall now analyse the non-equilibrium deviation *δW* = 〈*W*〉 − 〈*W*_ad_(*t*)〉 with respect to 〈*W*_ad_(*t*)〉. This is equivalent to the deviation of the mean energy of the motor along the super-adiabats from its (instantaneous) adiabatic expression. For a reversible isothermal process 〈*W*_ad_〉 = Δ*F* and *δW* = 〈*W*_irr_〉. Differently, for the adiabatic dynamics of stages 1 and 3 of the Otto cycle, conservation of the population in |*n*(*t*)〉 is satisfied provided that *β_t_* = *βε_n_*(0)/*ε_n_*(*t*), as it is the case for a large-class of self-similar processes, as discussed in Refs. 23,36,37 and remarked in the Supplementary Information. Here, *β_t_* is introduced by noticing that the physical adiabatic state at time *t* is characterised by the occupation probabilities 
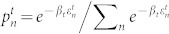
. Therefore, the reference state 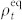 is not the physical instantaneous equilibrium state 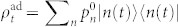 resulting from the adiabatic dynamics, and we find 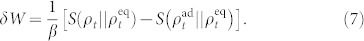
Therefore, in general *δW* ≠ 0. However, one can check that at the end of the process we have 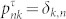, which implies *δW* = 0 and thus the frictionless nature of the process [cf. Fig. 2(c)]. The time-evolution of the different contribution to *δW*, *i.e.* 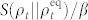 and 
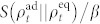
, are displayed in Fig. 2(d). This result is remarkable in the context of the quantum Otto cycle: If the baths are reconnected at time *τ* after both the compression and expansion stages, then the efficiency of an ideal reversible engine can be reached in finite-time, thus implementing a frictionless finite-time cycle. As friction is the only source of irreversibility in our scheme, the super-adiabatic engine reaches the maximum efficiency of an ideal quasi-static engine in a finite-time only.

**Figure 3:**
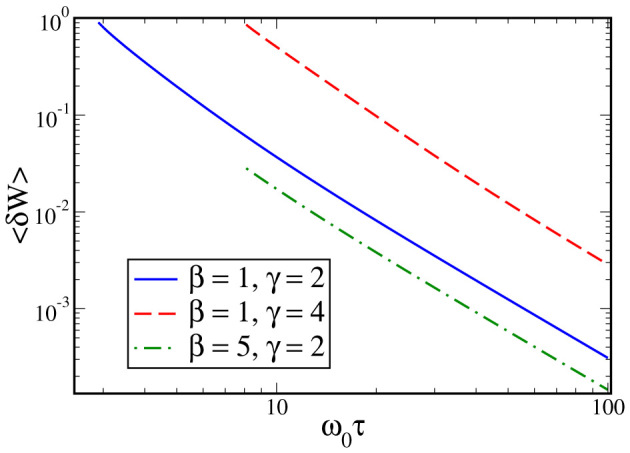
Quantum cost of running the super-adiabatic expansion stage of the quantum Otto cycle. We plot the time-averaged deviation 〈*δW*〉 of the mean energy of the system from the adiabatic eigen-energies for three values of *β* and 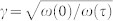. In all cases there is an effective power-law scaling of the form 〈*δW*〉 ~ 1/*τ*. The cut-off time is such that the confining potential remains a trap along the process, without the need for transiently inverting it to achieved the required speed up.

Let us address a final point: The efficiency in Eq. (1) diminishes with the breakdown of adiabaticity^13^. In contrast, our super-adiabatic engine achieves the maximum possible value 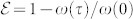. Clearly, if unlimited resources are available, there is no fundamental lower-bound on the running time of the adiabats. However, we take a pragmatic approach and quantify the energy cost associated with the implementation of our super-adiabatic engine, which would provide a significant cost function for such part of the cycle. We have thus considered the time-averaged dissipated work 
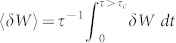
, ensuring *ω*^2^(*t*) > 0 for *t* ∈ [0, *τ*]. The cut-off time *τ_c_* was taken as the maximum running time along the shortcut of each super-adiabat before the trap is inverted (cf. Methods section). When this occurs, the adiabatic eigen-energies are not well defined and our formalism breaks down. For the shortcut to adiabaticity discussed here, such inversion occurs when the expansion time is smaller than the inverse of the initial frequency of the trap^36^. The exact value of such critical running time, which depends on the expansion factor and can be found numerically, is different for expansion and compression stages, being larger in the former case. While the steps necessary for the calculation of 〈*δW*〉 are reported in the Supplementary Information, here is enough to mention that the cost of running the super-adiabatic engine exhibits a 〈*δW*〉 ~ 1/*τ* behavior for a wide range of parameters, as shown in Fig. 3. This demonstrates the existence of a trade-off between the running time of the super-adiabatic transformations and the corresponding amount of time-averaged dissipated work, in line with the analogous compromise between the irreversible entropy produced along the isochores and the running time of the transformations. An upper bound for the power of an engine run can be calculated using the fundamental limitations set by quantum speed limit. The key steps of such calculations are discussed in the Supplementary Information.

## Discussion

We have demonstrated the possibility to perform a fully frictionless quantum cycle in a finite-time. Our proposal exploits the idea of shortcuts to adiabaticity, which allowed us to bypass the effects of friction on the compression and expansion stages in an important cycle such as Otto's. Our study embodies *one* example of the potential brought about by the combination of shortcuts to adiabaticity and the framework for out-of-equilibrium dynamics of a quantum system. The possibility to achieve maximum efficiency of a quantum engine at finite time with virtually no friction is tantalizing in the perspective of designing micro- and nano-scale motors operating at the verge of quantum mechanics.

## Methods

### Driving protocol of the super-adiabats

**Figure 4:**
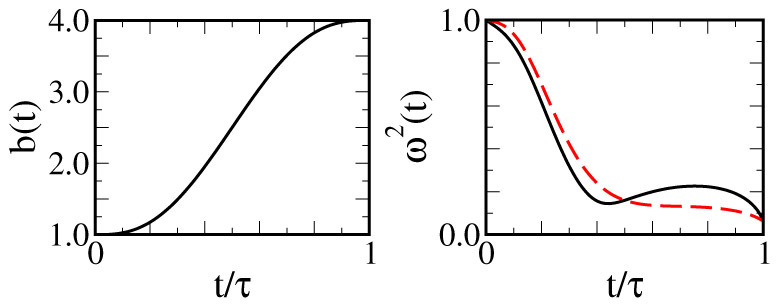
Scaling factor and driving frequency along a shortcut to an adiabatic expansion in a time-dependent harmonic trap. The scaling factor varies from *b*(0) = 1 to *b*(*τ*) = 4 in a time scale *τ* = 10/*ω*_0_ (solid line) and 20/*ω*_0_ (dashed line).

Here we illustrate the formal procedure for the determination of the scaling factor *b*(*t*) used in the super-adiabatic steps of our proposal. The simplest interpolation of the actual solution of the Ermakov equation in the main body of the paper with the boundary conditions stated in the main text is found to be the polynomial 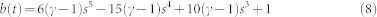
with *s* = *t*/*τ*. This solution guarantees that, in the adiabatic limit, *b*·(*t*) → 0 and *b*(*t*) → *b_ad_*, while more generally the eigenstates of the initial oscillator will evolve according to the scaling law in Eq. (4). The modulation of *ω*(*t*) is the responsible for the speed-up of the transformations performed along the super-adiabats. In turn, the implementation of such modulation is the price to pay for the achievement of such advantage. The explicit form *ω*(*t*) can be extracted from the Ermakov equation, 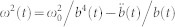, using Eq. (8) for *b*(*t*), see Fig. (4). For sufficiently small values of *ω*_0_*τ*, *ω*(*t*) can become purely imaginary (requiring the inversion of the trap into an expelling barrier). The condition *ω*^2^(*t*) > 0 provides the cut-off time *τ_c_* used in Fig. 3.

## Author Contributions

A.d.C. and J.G. developed the calculation for the concept proposed by M.P. All authors interpreted the results and wrote the manuscript.

## Supplementary Material

Supplementary Information Supplementary Information
