# Supplementary material for: More bang for your buck: Super-adiabatic quantum engines
Source: Sci Rep. 2014 Aug 28;4:6208. doi: 10.1038/srep06208 (PMC4147366; doi:10.1038/srep06208)
Supplement: Supplementary Information [file srep06208-s1.pdf]

Supplementary Information on "More bang for your buck: Super-adiabatic quantum engines"

A. del Campo,[1,2] J. Goold,[3,4] and M. Paternostro[5]

[1]Theoretical Division, Los Alamos National Laboratory, Los Alamos, NM 87545, USA
[2]Center for Nonlinear Studies, Los Alamos National Laboratory, Los Alamos, NM 87545, USA
[3]Clarendon Laboratory, Department of Physics, University of Oxford, Oxford OX1 3PU, United Kingdom
[4]The Abdus Salam International Centre for Theoretical Physics, 34014, Trieste, Italy
[5]Centre for Theoretical Atomic, Molecular and Optical Physics, School of Mathematics and Physics, Queen's University Belfast, BT7 1NN Belfast, United Kingdom

In this Supplementary Information document we provide a detailed analysis of some of the technical points of our investigation.

I. THERMALIZATION PROCESS DURING THE ISOCHORIC TRANSFORMATIONS

In this Section we study the thermalisation processes inherent in the isochoric transformations included in our engine cycle. The starting state of each transformation corresponds to a squeezed thermal state of the working medium (a harmonic oscillator), while the thermalization process itself can be modelled as relaxation induced by a bath at a given operating temperature. Our goal is to show that thermalisation is achieved within finite-time intervals and that the corresponding irreversible entropy produced across such relaxation can be kept at bay, and considered ineffective for the sake of determining the efficiency of the engine that we propose. In what follows, we will make use of the powerful formalism of covariance states, which is handy given the nature of the states and transformations at hand. The covariance matrix $\boldsymbol{\sigma}$ of a harmonic oscillator is defined as $\sigma_{ij}=\frac{1}{2}(\langle\{\hat{Q}_i,\hat{Q}_j\}\rangle-\langle\hat{Q}_i\rangle\langle\hat{Q}_j\rangle)$ with $\mathbf{Q}=(q,p)$ the vector of quadrature operators $\hat{q}=(\hat{a}+\hat{a}^\dagger)/\sqrt{2}$ and $\hat{p}=i(\hat{a}^\dagger-\hat{a})/\sqrt{2}$ of the oscillator and $\{\cdot,\cdot\}$ the anti-commutator. Here $\hat{a}$ ($\hat{a}^\dagger$) is the annihilation (creation) operator of a harmonic oscillator.

The covariance matrix of a single mode squeezed thermal state [1] can be straightforwardly obtained from using the relation $\mathscr{S}\sigma_{th}\mathscr{S}^{\mathsf{T}}$ with $\sigma_{th}=(2\overline{m}+1)\mathbb{1}_2$ the covariance matrix of a thermal state of mean occupation number $\overline{m}$ ($\mathbb{1}_2$ is the 2×2 identity matrix) and

$$\mathscr{S}=\begin{pmatrix} e^{-r} & 0\\ 0 & e^{r}\end{pmatrix} \tag{1}$$

the linear canonical transformation corresponding to the squeezing operation $e^{\frac{r}{2}(\hat{a}^{\dagger 2}-\hat{a}^2)}$ with r is the squeezing factor.

A harmonic oscillator that is in contact with a thermal bath at inverse temperature β_b such that $\overline{n}=(e^{\beta_b\omega}-1)^{-1}$ evolves according to the master equation

$$\partial_t\rho=\frac{\gamma}{2}[(\overline{n}+1)(2\hat{a}\rho\hat{a}^\dagger-\{\hat{a}^\dagger\hat{a},\rho\})+\overline{n}(2\hat{a}^\dagger\rho\hat{a}-\{\hat{a}\hat{a}^\dagger,\rho\})] \tag{2}$$

with ρ the density matrix of the oscillator and γ the oscillator energy damping rate. This equation, which is valid in the limit of weak-coupling between the oscillator and its environment, can be solved using phase-space methods leading to the following time evolved covariance matrix [2]

$$\begin{aligned}\sigma_{th}(t)&=(2\overline{n}+1)(1-e^{-\gamma t})\mathbb{1}_4+\sigma_{th}e^{-\gamma t}\\ &=\begin{pmatrix} e^{-2r-t\gamma}(2\overline{m}+1)+(1-e^{-t\gamma})(2\overline{n}+1) & 0\\ 0 & e^{2r-t\gamma}(2\overline{m}+1)+(1-e^{-t\gamma})(2\overline{n}+1)\end{pmatrix}.\end{aligned} \tag{3}$$

Our goal here is to estimate the time t needed by the oscillator to relax towards a thermal state with a mean phonon number $\overline{n}$. Our figure of merit in this respect is embodied by the fidelity between the state characterised by the covariance matrix Eq. (3) and the thermal state with covariance matrix $\sigma_b=(2\overline{n}+1)\mathbb{1}_4$. As we are dealing with Gaussian states and processes, we can write such fidelity in terms of the respective covariance matrices only as [3]

$$F(\sigma_{th}(t),\sigma_b)=\frac{2}{\sqrt{\Delta+\Lambda}-\sqrt{\Lambda}} \tag{4}$$

with

$$\begin{aligned}\Delta&=\det[\sigma_{th}(t)+\sigma_b],\\ \Lambda&=(\det[\sigma_b]-1)(\det\sigma_{th}(t)-1).\end{aligned} \tag{5}$$

The analytic expression for the fidelity enables the calculation of a lower bound to the irreversible entropy $\Delta S^{\rm th}_{\rm irr}$ produced during the thermalisation process, which is evaluated by following the formal apparatus presented in Ref. [4] according to which $\Delta S^{\rm th}_{\rm irr}\geq B(t)=s[2\mathscr{L}(\sigma_{th}(t),\sigma_b)/\pi]$ with

$$s[x]=2x^2+4x^4/9+32x^6/135+\mathscr{O}(x^8) \tag{6}$$

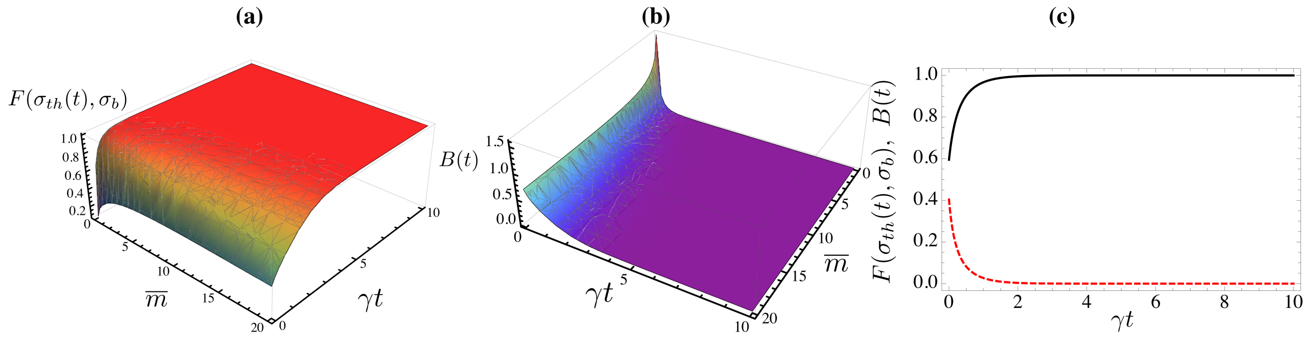

FIG. 1: Fidelity $F\sigma_{th}(t),\sigma_b)$ [panel **(a)**] and bound to the irreversible entropy $B(t)$ [panel **(b)**] plotted against the dimensionless system-bath interaction time γt and the initial phonon occupation number $\overline{m}$ of the working medium for $\overline{n}=10$ and $r=1$. Panel **(c)** shows a comparison between these two functions (the fidelity [irreversible entropy] being represented by the solid black line [dashed red line]) for $\overline{m}=5$. These behaviours should be taken as typical.

and $\mathscr{L}(\sigma_{th}(t),\sigma_b)=\arccos\sqrt{F(\sigma_{th}(t),\sigma_b)}$ the Bures angle between the states defined by their respective covariance matrix. Both the fidelity and the bound to the irreversible entropy are shown in Fig. 1 **(a)** and **(b)** for a choice of the initial degree of squeezing of the oscillator and temperature of the bath. As we are interested in the time taken by the oscillator to thermalise to the bath it is in contact with, we study such functions against the dimensionless oscillator-bath interaction time γt and the initial value of $\overline{m}$. The behaviors shown in Fig. 1 should be taken as typical, as verified by exploring these function within a wide range of values of the involved parameters. Clearly, a working point exists such that the system equilibrates within a time $\gamma t^*\simeq 1$ [such time is determined by considering the threshold value of γt at which $F(\sigma_{th}(t^*),\sigma_b)\gtrsim 0.9$]. Although $B(t)$ embodies only a lower bound, we have checked that within the range of parameters used in Figs. 1 **(a)**-**(c)**, it is faithful to the explicit evaluation of the entropy produced in the process according to the general (and much more involved) approach put forward in Ref. [5], and involving the quantum Gaussian relative entropy between the initial squeezed thermal state and the final equilibrium one at the temperature of the bath. As such, in what follows we stick to the use of $B(t)$ as providing a reliable and easily grasped estimate of the irreversible entropy produced in the system, which is kept at quite low levels. A comparison between $F(\sigma_{th}(t),\sigma_b)$ and the bound, here labelled $B(t)$ (we omit its explicit form for simplicity), is given in Fig. 1 **(c)** for $\overline{m}=\overline{n}/2=5$. Needless to say, as this analysis is valid for $\gamma\ll\omega(0),\omega(\tau)$ (for the validity of weak-coupling assumptions), these results should be kept in consideration when evaluating the power of the engine at hand.

This analysis shows that the running times $\tau_{2,4}$ of the isochores needed for the Otto cycle can be kept at finite values, still achieving effective low-entropy thermalisation processes that would leave Eqs. (1) and (2) of the main paper valid.

II. NONEQUILIBRIUM WORK FLUCTUATIONS

We next present the derivation of Eq. (7) in the main paper. Consider as reference states the fictitious equilibrium state $\rho_t^{\rm eq}$ and the adiabatic one $\rho_t^{\rm ad}$. Then, in the adiabatic limit the average work can be rewritten as

$$\begin{aligned}\langle W_{\rm ad}(t)\rangle &= {\rm Tr}[\rho_0(\hat{\mathscr{H}}(t)-\hat{\mathscr{H}}(0)]=\sum_{\rm n}{\rm p}_{\rm n}^0[\varepsilon_{\rm n}(t)-\varepsilon_{\rm n}(0)]\\ &= -\frac{1}{\beta}\sum_n p_n^0 \ln p_n^t+\frac{1}{\beta}\sum_n p_n^0\ln p_n^0-\frac{1}{\beta}\ln(Z_t/Z_0)\\ &= \frac{1}{\beta}S(\rho_t^{\rm ad}||\rho_t^{\rm eq})+\Delta F \end{aligned}\tag{7}$$

with $Z_t={\rm Tr}[{\rm e}^{-\beta\hat{\mathscr{H}}(t)}]$ the instantaneous partition function. For a general nonequilibrium process, the average work reads instead

$$\begin{aligned}\langle W\rangle &= -\frac{1}{\beta}\sum_{nk}p_n^0p_{nk}^t\ln p_k^t+\frac{1}{\beta}\sum_n p_n^0\ln p_n^0-\frac{1}{\beta}\ln(Z_t/Z_0)\\ &= \frac{1}{\beta}S(\rho_t||\rho_t^{\rm eq})+\Delta F.\end{aligned}\tag{8}$$

As a result, nonequilibrium deviations from the mean adiabatic work take the form

$$\delta W=\frac{1}{\beta}[S(\rho_t||\rho_t^{\rm eq})-S(\rho_t^{\rm ad}||\rho_t^{\rm eq})].\tag{9}$$

It is worth considering an an alternative approach, where $\rho_t^{\rm ad}$ is used as a reference state and the dynamics is restricted to the class of self-similar processes [6–8], for which conservation of the population in the mode $|n(t)\rangle$ as a function of time t is satisfied provided that

$$\beta_t=\beta\varepsilon_n(0)/\varepsilon_n(t),\tag{10}$$

as it is the case for the adiabatic dynamics associated to the shortcuts discussed here. Under such condition the partition of the instantaneous equilibrium state remains constant $Z_t=Z_0=Z$. Using $\rho_t^{\rm ad}$, the average work in the adiabatic limit reads

$$\begin{aligned}\langle W_{\rm ad}(t)\rangle &= \frac{1}{\beta}\sum_n p_n^0\ln p_n^0-\frac{1}{\beta_t}\sum_n p_n^0\ln p_n^t-(\frac{1}{\beta_t}-\frac{1}{\beta})\ln Z\\ &= \frac{1}{\beta_t}S(\rho_t)-\frac{1}{\beta}S(\rho_0)+\Delta F,\end{aligned}\tag{11}$$

where we have introduced the von Neuman entropy $S(\rho) = -\mathrm{Tr}[\rho \ln \rho]$ of an arbitrary state ρ. More generally,

$$\begin{aligned} \langle W \rangle &= \frac{1}{\beta}\sum_n p_n^0 \ln p_n^0 - \frac{1}{\beta_t}\sum_{k,n} p_{nk}^t p_n^0 \ln p_k^0 - (\frac{1}{\beta_t} - \frac{1}{\beta})\ln Z \\ &= \frac{1}{\beta_t} S(\rho_t) - \frac{1}{\beta} S(\rho_0) + \frac{1}{\beta_t} S(\rho_t || \rho_t^{\mathrm{ad}}) + \Delta F. \end{aligned} \quad (12)$$

This leads to the following compact expression for nonequilibrium work deviations form the adiabatic path,

$$\delta W = \frac{1}{\beta_t} S(\rho_t || \rho_t^{\mathrm{ad}}). \quad (13)$$

The two expressions for δW, Eqs. (9) and (13), agree for self-similar processes and vanish at the end of the stroke (either 1 or 3 in Fig. 1 of the main paper) both for a shortcut and in the adiabatic limit.

III. UPPER BOUND TO POWER THROUGH THE QUANTUM SPEED LIMIT

The quantum speed limit for a driven quantum system [9] allows us to derive an upper bound for the power of the engine. For simplicity, we can consider a equal-time shortcuts along the two super-adiabats so that $\tau = \tau_1 = \tau_3$. Then, it follows that

$$P \leq -\frac{\langle W_{\mathrm{ad},1}(\tau)\rangle + \langle W_{\mathrm{ad},3}(\tau)\rangle}{\hbar \mathscr{L}\left(\rho_\tau^{\mathrm{eq}}, \rho_0\right)} \max\{E_\tau, \Delta E_\tau\}. \quad (14)$$

where $E_\tau = \tau^{-1}\int_0^\tau dt \mathrm{Tr}[\rho_t \mathscr{H}(\mathrm{t})]$ with respect to the ground state energy, $\Delta E_\tau = \tau^{-1}\int_0^\tau dt \{\mathrm{Tr}[\rho_t \mathscr{H}^2(\mathrm{t})] - \mathrm{Tr}[\rho_t \mathscr{H}(\mathrm{t})]^2\}^{1/2}$, and the angle in Hilbert space between initial and target states is

$$\mathscr{L}\left(\rho_0, \rho_\tau^{\mathrm{eq}}\right) = \arccos\left(\sqrt{F\left(\rho_0, \rho_\tau^{\mathrm{eq}}\right)}\right) \quad (15)$$

in terms of the fidelity $F\left(\rho_0, \rho_\tau^{\mathrm{eq}}\right) = \left[\mathrm{Tr}\sqrt{\sqrt{\rho_0}\rho_\tau^{\mathrm{eq}}\sqrt{\rho_0}}\right]^2$. In a super-adiabatic engine, $\langle W \rangle_{\mathrm{ad},1} + \langle W \rangle_{\mathrm{ad},3}$ equals

$$\sum_{j=1,3} \langle W_{\mathrm{ad,j}}(\tau)\rangle = \frac{\hbar}{2}(\omega_0 - \omega_\tau)\left[\coth\frac{\beta_c \hbar \omega(\tau)}{2} - \coth\frac{\beta \hbar \omega_0}{2}\right] \quad (16)$$

where β_c is the inverse temperature of the cold bath during stage 2.

[1] A. Ferraro, S. Olivares, and M. G. A. Paris, Gaussian States in Quantum Information, Bibliopolis, Napoli, 2005.
[2] M. Paternostro, G. Adesso, and S. Campbell, Phys. Rev. A **80**, 062318 (2009).
[3] P. Marian, and T. A. Marian, Phys. Rev. A **86**, 022340 (2012); H. Scutaru, J. Phys. A: Math. Gen. **31**, 3659 (1998); Gh.-S. Părăoanu and H. Scutaru, Phys. Rev. A **58**, 869 (1998)
[4] S. Deffner, and E. Lutz, Phys. Rev. E **87**, 022143 (2013).
[5] S. Deffner, and E. Lutz, Phys. Rev. Lett., **107**, 140404 (2011).
[6] X. Chen, *et al.*, Phys. Rev. Lett. **104**, 063002 (2010).
[7] A. del Campo, Phys. Rev. A, **84**, 031606(R) (2011).
[8] A. del Campo and M. G. Boshier, Sci. Rep. **2**, 648 (2012).
[9] S. Deffner and E. Lutz, J. Phys. A: Math. Theor. **46**, 335302 (2013).
